# Environmental Change and Disease Dynamics: Effects of Intensive Forest Management on Puumala Hantavirus Infection in Boreal Bank Vole Populations

**DOI:** 10.1371/journal.pone.0039452

**Published:** 2012-06-20

**Authors:** Liina Voutilainen, Sakeri Savola, Eva Riikka Kallio, Juha Laakkonen, Antti Vaheri, Olli Vapalahti, Heikki Henttonen

**Affiliations:** 1 Finnish Forest Research Institute, Vantaa Research Unit, Vantaa, Finland; 2 Department of Virology and Research Programs Unit, Infection Biology, Haartman Institute, University of Helsinki, Helsinki, Finland; 3 Department of Biological and Environmental Science, University of Jyväskylä, Jyväskylä, Finland; 4 Department of Evolution, Ecology and Behaviour, Institute of Integrative Biology, University of Liverpool, Liverpool, United Kingdom; 5 Department of Veterinary Biosciences, Faculty of Veterinary Medicine, University of Helsinki, Helsinki, Finland; 6 Helsinki University Central Hospital, HUSLAB, Helsinki, Finland; Duke-NUS Gradute Medical School, Singapore

## Abstract

Intensive management of Fennoscandian forests has led to a mosaic of woodlands in different stages of maturity. The main rodent host of the zoonotic Puumala hantavirus (PUUV) is the bank vole (*Myodes glareolus*), a species that can be found in all woodlands and especially mature forests. We investigated the influence of forest age structure on PUUV infection dynamics in bank voles.

Over four years, we trapped small mammals twice a year in a forest network of different succession stages in Northern Finland. Our study sites represented four forest age classes from young (4 to 30 years) to mature (over 100 years) forests. We show that PUUV-infected bank voles occurred commonly in all forest age classes, but peaked in mature forests. The probability of an individual bank vole to be PUUV infected was positively related to concurrent host population density. However, when population density was controlled for, a relatively higher infection rate was observed in voles trapped in younger forests. Furthermore, we found evidence of a “dilution effect” in that the infection probability was negatively associated with the simultaneous density of other small mammals during the breeding season.

Our results suggest that younger forests created by intensive management can reduce hantaviral load in the environment, but PUUV is common in woodlands of all ages. As such, the Fennoscandian forest landscape represents a significant reservoir and source of hantaviral infection in humans.

## Introduction

Human activity has changed the distribution and abundance of many species. Consequently, the distribution and abundance of pathogens and parasites that these species harbor may also be altered. Several wildlife pathogens can also infect humans, i.e., are zoonotic. For example, hantaviruses (family *Bunyaviridae*) borne by rodents and insectivores cause hemorrhagic fever with renal syndrome (HFRS) and hantavirus cardiopulmonary syndrome (HCPS) in humans [1].

The most common hantaviral disease in Europe is nephropathia epidemica (NE), a mild form of HFRS [2]. It is caused by Puumala virus (PUUV) [3], [4] which is typically found in the bank vole, *Myodes glareolus*. In the rodent host, PUUV is persistent [5] and does not cause visible symptoms [5]–[7] although it can reduce winter survival [8]. Transmission in the host is horizontal and occurs directly and via aerosol excretions [6], [9]. The latter is considered the main pathway for rodent-human transmissions [10]. Epidemic peaks of human NE follow the seasonal and multiannual dynamics of bank vole abundance [11]–[18]. While the virus and its host are found in most European countries, the bulk of human infections occur in the boreal zone of North and North-east Europe [2].

Areas of intensive agriculture have been associated with lower prevalence of PUUV in bank voles [19]–[21] and lower incidence of NE [14], [20], [22] than in more forested areas in the temperate zone. In this study, we investigated whether another kind of environmental disturbance, i.e., the intensive forestry practiced in boreal Fennoscandia could have similar effects on PUUV dynamics. Intensive management of Fennoscandian forests has transformed the landscape into a mosaic of grassy clear-cuts, plantations, and a succession of forests of variable maturity. Forest cutting profoundly alters ground-level vegetation, when mosses and *Vaccinium* shrubs of mature forest floors are replaced by grasses in newly cut sites undergoing reforestation [23], [24]. In boreal Finland, land area is nearly 80% forested, of which the vast majority is in commercial production. Climax forests (100+ years) represent approximately 20% of managed forests, whereas the proportion of young stands (0–40 years) is roughly twice as large [25].

Dearing and Dizney [26] reviewed the mechanisms by which environmental disturbance can influence the infection dynamics of hantavirus via their rodent hosts. Firstly, disturbance can alter host population density, which in turn may affect intraspecific contact rates. Intensive management of boreal forests can reduce bank vole abundance since they prefer climax forests, probably due to their richness in food sources (bilberry, arboreal lichens) and shelter (moss, woody debris) [27]–[29]. Another suggested driving force, the so-called “dilution effect” [30]–[32], proposes that habitat disturbance can alter small mammal species richness, influencing intraspecific contact rate and thereby transmission [26]. In boreal environments, small mammal communities are often more diverse in young managed forests as this is the preferred habitat of *Microtus* spp. [27].

In this study, we investigated whether intensive management of boreal forests influences the dynamics of PUUV in bank voles. Given their preference for mature forests, we expected the abundance of PUUV-infected bank voles to be positively related to forest age. Furthermore, we expected bank vole density to be positively related and the abundance of non-host species to be negatively related to the probability of bank voles being PUUV infected. To test these predictions, we monitored small mammal species abundance and the incidence of PUUV-infected bank voles in four forest age classes over four years in northern Finland. With this design, we were able to explore the relationships between forest age, small mammal community structure and infection dynamics of PUUV in bank voles.

## Materials and Methods

### Ethics Statement

According to the Finnish Act on the Use of Animals for Experimental Purposes (62/2006) and a further decision by the Finnish Animal Experiment Board (16^th^ May, 2007), the animal capture technique, i.e., using traps that instantly kill the animal, we used is not considered an animal experiment and therefore requires no animal ethics license from the Finnish Animal Experiment Board. All animal trapping took place with permission (1013/204/2002) on land owned by the Finnish Forest and Park Service. A permit (23/5713/2001) for capturing protected species (*Sorex* spp., *Myodes rufocanus* and *Myopus schisticolor*) was granted by the Finnish Ministry of the Environment. Other species captured in this study are not protected in Finland and none of the captured species are included in the Red List of Finnish Species.

### Study Site and Design

The study was conducted within an area of 875 km^2^ in northern Finland (municipalities of Taivalkoski and Pudasjärvi: 65° N, 28°E) in the northern boreal vegetation zone. Management of each forest stand was recorded in detail by the Finnish Forest and Park Service and included dates on which clear-cutting and plantation took place.

Stands in our study represented four age classes: managed forests with (1) 4–8, (2) 10–15, and (3) 25–30 years from plantation after clear cutting, and non-managed forests (4) older than 100 years (see Figure S1 for representative photographs). In each age class, 10 forest sites of 5–76 ha (average 18 ha) were chosen for the study. Forest stands >100 years were dominated by Norwegian spruce (*Picea abies*), whereas the younger forests were plantations of spruce or Scots pine (*Pinus sylvestris*) with some naturally regenerated birch (*Betula* spp.), aspen (*Populus tremula*) and rowan (*Sorbus aucuparia*). In the understory vegetation, mosses (*Hylocomium splendens*, *Pleurozium schreberi* and *Dicranum* spp.) together with bilberry (*Vaccinium myrtillus*) were dominant in mature forests. In 4–8 year forest stands, mosses were replaced by grasses (mostly *Deschampsia flexuosa*), fireweed (*Epilobium angustifolium*) and raspberry (*Rubus idaeus*). Grasses were still abundant 10–15 years after cutting, but less abundant than in the youngest age class. Shrubs such as *Vaccinium vitis-idaea*, *Empetrum nigrum*, *Vaccinium uliginosum* and *Rhododendron tomentosum* were common. In 25–30 year stands, much of the ground was shaded resulting in sparse understory vegetation than in other age classes. Grasses were rare, and some *V. vitis-idaea* and *V. myrtillus* were present.

### Small Mammal Trapping

Small mammals were trapped according to the small quadrat method [33]. Within each stand, small mammals were trapped in five 15×15 m quadrats that were accessible by foot but at least 50 m apart. Twelve snap-traps were baited with rye bread and placed on each quadrat so that three traps were placed within 2 m from each corner. Traps were set over two consecutive nights and checked each morning. Trappings were performed on 200 quadrats on 40 stands during the beginning (early June) and end (September) of the breeding season from 2007 to 2010. Each trapping period lasted approximately three weeks.

Once trapped, small mammals were placed in plastic bags in cooled, insulated containers and frozen the same day at −20°C. Animals were later dissected and their species, sex, age, and weight determined. Age determination of bank voles (summer-born/overwintered) was based on the development of pelage, and when uncertain, on molar roots [34]. The heart of each bank vole was placed in an Eppendorf vial and kept frozen until screened for the presence of PUUV antibodies. PUUV antibodies are preserved in heart samples as well as in fresh-frozen whole blood samples (unpublished data).

### Determination of PUUV Infection

The heart of each animal was diluted in 200 µL phosphate-buffered saline (PBS) prior to an immunofluorescent antibody test (IFAT) to detect PUUV-specific antibodies [35]. PUUV-infected female bank voles provide maternal antibodies to their offspring [6], [36] which may inflate the infection rate in such cases. Following Kallio and others [37], we expected the proportion of seropositive individuals to decrease with body mass until a certain threshold where maternal antibodies are commonly lost, and then to increase again with body mass, since older and heavier bank voles are often PUUV infected [37]–[39]. To determine the threshold between PUUV infection and seropositivity due to maternal antibodies, we applied a generalized additive mixed model (GAMM) with binomial error distributions and a logit link function (gamm4 function of gamm4 package [40] in the R software package [41]). A body mass smoother was used as predictor for the probability of a bank vole being PUUV seropositive, and a random intercept was allowed for each stand and trapping session. As summer-born animals were only captured in the fall, animals captured in spring were excluded from the data set.

### Statistical Analysis

The effect of forest age on the number of PUUV-infected bank voles per stand was studied using GLMM (generalized linear mixed modeling) with Poisson error distributions and a log link function. In the full model, forest age (factor with four levels), vole density cycle phase (high; years 2007 and 2010/low; years 2008 and 2009), season (spring/fall), and all their two-way interactions were used as fixed factors. To account for repeated measurements, a random intercept was allowed for each forest stand, and to account for overdispersion, for each observation [42]. The full model was reduced by removing fixed effects sequentially if their inclusion did not decrease AIC (Akaike information criterion) by more than two units [43].

The probability of a bank vole being infected with PUUV was studied using GLMM with binomial error distributions and a logit link function. Forest age (factor with four levels) and the mean-centered numbers of bank voles and other small mammals detected were included as predictors. Furthermore, to exclude the observed effects that would result from demographic variation in bank vole populations (i.e., confounding effects), we added the mean-centered body mass as a proxy of age, sex, and their interaction as predictors. To account for repeated measurements, random intercepts were allowed for each forest stand and trapping session. As we expected the ranges of both the dependent and independent variables to vary between seasons, separate models were applied for spring and fall data. Inferences were drawn from the full models, since several independent variables (e.g., the number of bank voles and other small mammals) were expected to confound each other. All models were fitted using the Laplace approximation method (lmer function of lme4 package [44] in the R software [41]).

## Results

**Figure 1:**
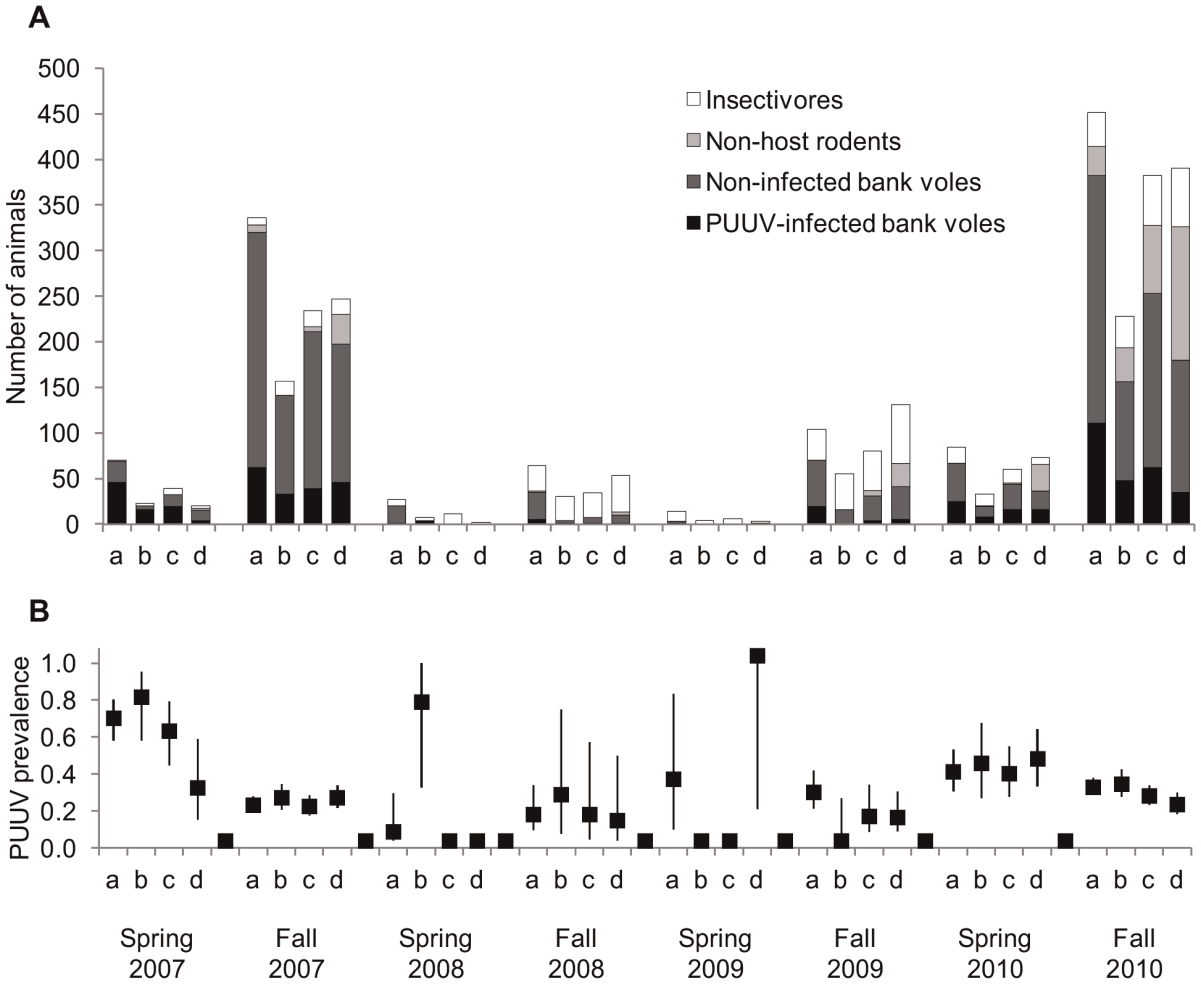
Numbers of small mammals (A) and Puumala hantavirus prevalence (B) in four forest succession stages. a = 100+; b = 25–30; c = 10–15; d = 4–8 years after planting.

**Figure 2:**
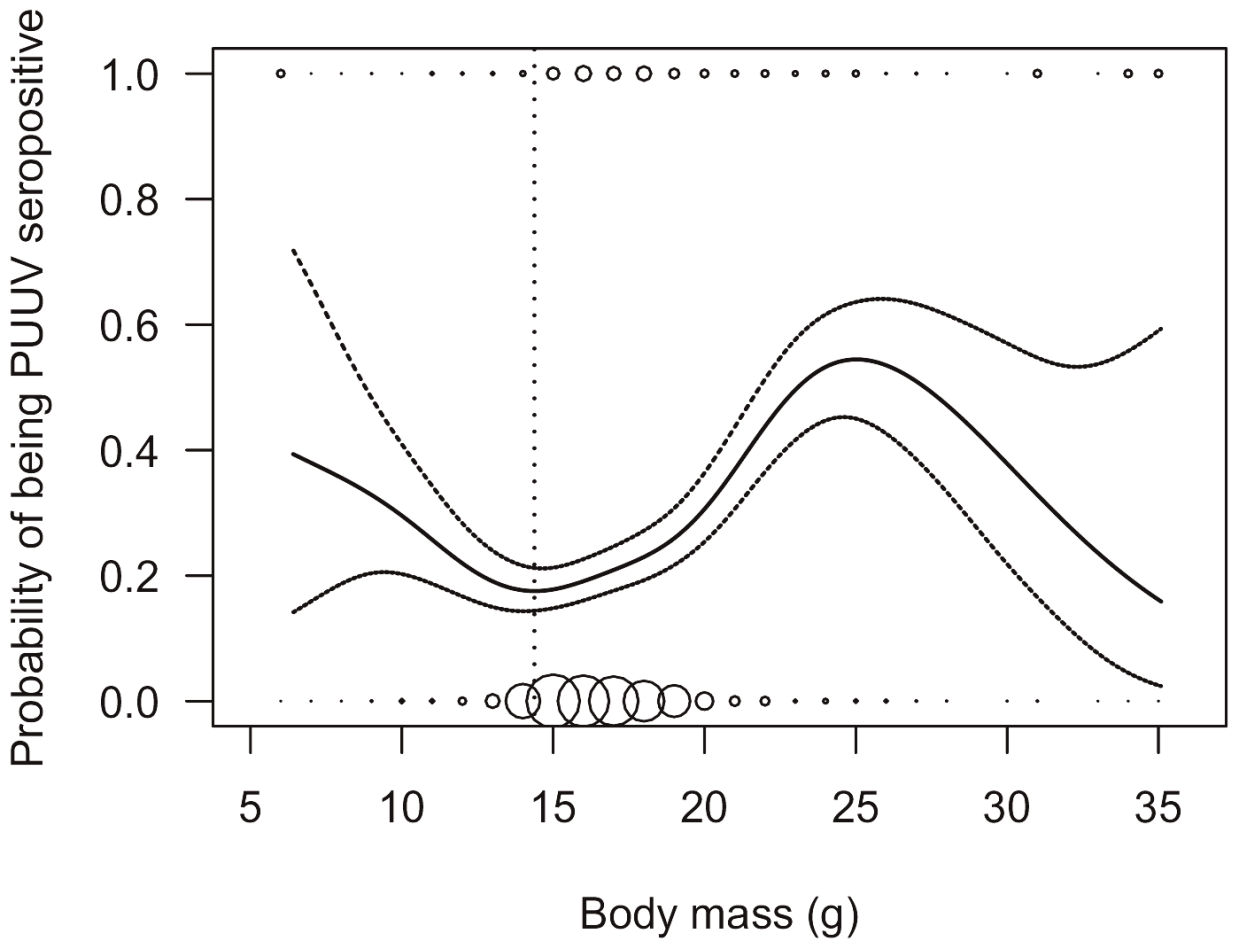
Differentiation between Puumala hantavirus infection and maternal antibodies. Predicted probability (solid line) with 95% CI (dashed lines) of bank voles captured in fall to carry hantavirus antibodies in relation to body mass. The dotted vertical line indicates the threshold value (14.4 g) above which seropositive animals were treated as genuinely infected. Spheres indicate the numbers of observed data points.

Altogether, 3 452 small mammals were captured during eight trapping periods from June 2007 to September 2010. The most common species was the bank vole (N = 2 384, Figure 1A). Four other rodent species (field vole *Microtus agrestis* (N = 326), root/tundra vole *Microtus oeconomus* (N = 25), wood lemming *Myopus schisticolor* (N = 33), gray-sided vole *Myodes rufocanus* (N = 21)), and four shrew (Soricinae) species (common shrew *Sorex araneus* (N = 624), masked shrew *Sorex caecutiens* (N = 34), pygmy shrew *Sorex minutus* (N = 3), and water shrew *Neomys fodiens* (N = 2)) were also captured. Bank voles were most abundant in >100 year forests, whereas non-host rodents (i.e., pooled numbers of field voles, root voles, grey-sided voles and wood lemmings) were most abundant in the 4–8 year forests. Shrew abundance did not show distinct differences between forest age classes (Fig. 1A). All bank voles captured in spring had over-wintered and were reproductive, whereas most in the fall were non-breeding individuals born during the summer. From the springtime trapping periods, 148 of the 318 bank voles screened carried PUUV antibodies and were considered infected (46.5%, Wald’s 95% CI: 41.1 to 52.0%). From the fall trapping periods, 513 of 1 947 bank voles (26.3%) screened positive for PUUV-specific antibodies. The probability of carrying antibodies in the fall reached its minimum at a weight of 14.4 grams (Figure 2). Thus, in later analyses seropositive bank voles with a body mass <14.4 g (N = 67) were considered to be carrying maternal antibodies and therefore not infected. Consequently, infection prevalence in the fall trapping periods was 22.9% (95% CI: 21.1 to 24.8%). There were no discernable differences in PUUV infection prevalence between the four forest age classes examined (Figure 1B).

### Effect of Forest Age on the Abundance of PUUV-infected Bank Voles

PUUV-infected bank voles were most abundant in 100+ year forests (Figure 1A). The best-supported Generalized Linear Mixed Model (GLMM) analyzing the abundance of PUUV-infected bank voles included the main effects of forest age, season, and cycle phase (Table 1), but all their two-way interactions were dropped during model selection. Compared to 100+ year forests, the predicted abundance of infected bank voles in 25–30, 10–15, and 4–8 year forests was 61%, 46%, and 65% lower, respectively (transformed from GLMM coefficients in Table 1 by coefficient(%)  =  [1-e^Intercept +^^Coefficient^/e^Intercept^]*100). All these differences were statistically significant. Furthermore, PUUV-infected voles were 71% less abundant in spring than fall and 95% less abundant during low density years.

**Table 1:** Best supported GLMM analyzing the number of PUUV-infected bank voles in a forest stand.

Parameter	Estimate (SE)^a^	*z*	P value
Intercept^b^	2.062 (0.203)	10.2	<0.0001
Forest age (25–30 y)	−0.945 (0.285)	−3.3	0.00093
Forest age (10–15 y)	−0.621 (0.279)	−2.2	0.02577
Forest age (4–8 y)	−1.039 (0.288)	−3.6	0.00031
Cycle phase (low)	−2.906 (0.209)	−13.9	<0.0001
Season (spring)	−1.250 (0.145)	−8.7	<0.0001

Variance attributable to random forest stand effect (40 groups) was 0.23 with standard deviation 0.48 and to random observation-level effect (320 groups) 0.44 with standard deviation 0.66.

a Estimates are given on natural logarithmic scale and standard errors of estimates are in parentheses.

b Intercept is calculated for a 100+ year stand in the fall of a high density year.

### Effects of Forestry, Bank Vole Density and Non-host Species on PUUV Infection in Bank Voles

**Figure 3:**
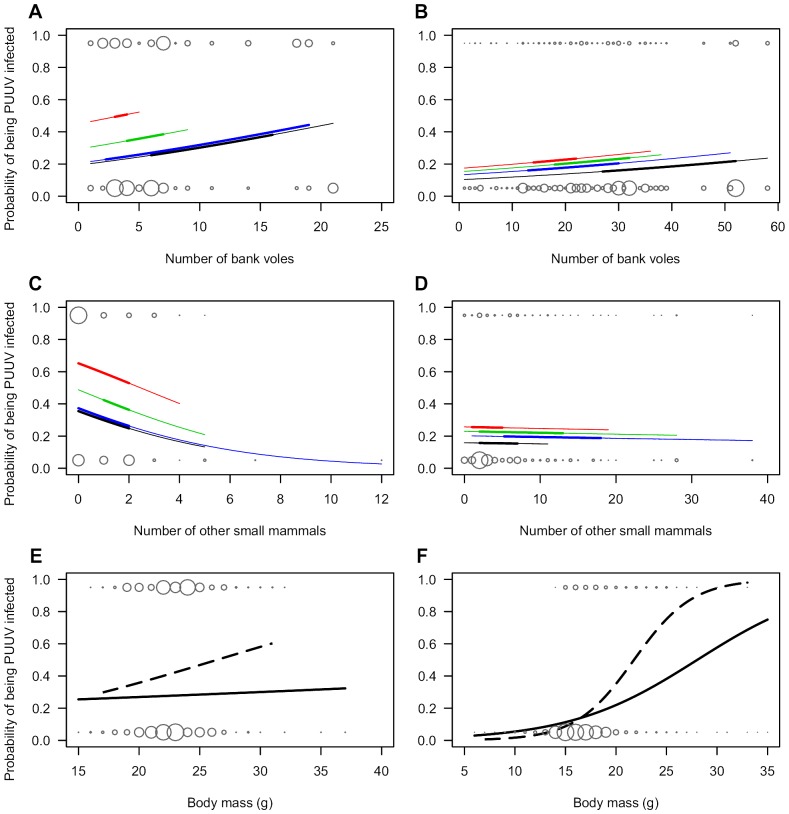
Factors contributing to the probability of a bank vole being infected by Puumala hantavirus. Predicted probabilities of a bank vole being PUUV infected in relation to forest age and concurrent numbers of bank voles on the study site in spring (**A**) and fall (**B**), in relation to forest age and concurrent numbers of other small mammals in (**C**) spring and (**D**) fall, and in relation to sex and body mass in spring (**E**) and fall (**F**). Predicted values represent estimates of fixed effects in Table 2. In **A, B, C,** and **D,** black line = 100+; red = 25–30; green = 10–15; blue = 4–8 year forest stand; all predictions are made for a bank vole female with average values of other continuous covariates. In **E** and **F,** solid lines indicate predictions for females, and dashed lines for males, both captured from a 100+ year stand with average numbers of bank voles and other small mammals. Spheres indicate the numbers observed, sphere sizes between plots are not comparable. Predictions are only drawn on the range of observations on each forest age class (**A,B,C,D**) and sex (**E,F**). Thick lines in **A,B,C,** and **D** represent the interquartile ranges of observations.

Bank voles in young forests were more likely to be PUUV infected than those in old forests (Figure 3A–D, Table 2). In the GLMM the probability of a bank vole being PUUV infected in spring was significantly lower in 100+ than 25–30 year forests. In the fall model, the difference was also significant between animals in 100+ and 10–15 year forests. The probability of infection followed the same pattern in spring and fall, i.e., 25–30>10–15>4–8>100+ year forests (Figure 3A–D, Table 2). The probability of PUUV infection increased with bank vole abundance, the effect being more pronounced in spring than in fall, but statistically significant in both seasons (Figure 3A–B, Table 2). The abundance of other small mammals had a statistically significant and negative impact on the probability of a bank vole being PUUV infected in spring, but not in fall (Figure 3C–D, Table 2). In spring, males were significantly more likely to be infected than females but the body weight and the interaction of sex and weight were not significant (Figure 3E, Table 2). In fall, both body weight and the interaction of sex and weight were significant predictors of infection status, so that the probability increased by weight more steeply in males than females (Figure 3F, Table 2). The model coefficients (Table 2) represent the effects of independent variables “all other things equal”, so that the probability of PUUV infection is higher in young compared to old forests at any given host or non-host density (Figure 3A–D). Likewise, in Figures 3A–D the predicted probabilities represent an average-weight female, whereas probabilities for males and heavier individuals would be higher in any given forest age class, or density of bank voles or other small mammals. Despite the fact that bank voles were more often infected in young forests, their higher density in old forests compensated, at least in part, for the habitat-attributed decrease in infection rate (Figure 3A–B). All correlations between fixed covariates were lower than 0.6, i.e., no collinearity was found that would have prevented their inclusion in the same model.

**Table 2:** GLMMs analyzing the probability of a bank vole being PUUV infected in spring and fall.

	Spring season			Fall season		
Parameter	Estimate^a^	z	P	Estimate^a^	z	P
Intercept^b^	**−0.957 (0.387)**	**−2.5**	**0.013**	**−1.705 (0.149)**	**−11.4**	**<0.0001**
Forest age (25–30 y)	**1.225 (0.454)**	**2.7**	**0.007**	**0.607 (0.221)**	**2.7**	**0.006**
Forest age (10–15 y)	0.547 (0.382)	1.4	0.152	**0.458 (0.197)**	**2.3**	**0.020**
Forest age (4–8 y)	0.079 (0.421)	0.2	0.852	0.296 (0.223)	1.3	0.185
N of bank voles	**0.059 (0.026)**	**2.3**	**0.023**	**0.017 (0.005)**	**3.3**	**0.001**
N of other small mammals	**−0.256 (0.109)**	**−2.4**	**0.019**	−0.005 (0.009)	−0.6	0.580
Sex (male)	**0.626 (0.257)**	**2.4**	**0.015**	0.158 (0.122)	1.3	0.196
Weight	0.015 (0.057)	0.3	0.790	**0.158 (0.019)**	**8.1**	**<0.0001**
Sex (male):Weight	0.076 (0.082)	0.9	0.354	**0.188 (0.039)**	**4.8**	**<0.0001**

In the spring, variance attributable to random forest pattern effect (39 groups) was 0.10 with standard deviation 0.31 and to random year effect (4 groups) 0.22 with standard deviation 0.47.

In the fall, variance attributable to random forest pattern effect (40 groups) was 0.05 with standard deviation 0.21 and to random year effect (4 groups) 0.00 with standard deviation 0.00.

a Estimates are given on logit scale and standard errors of estimates are in parentheses. Significant coefficients are in bold.

b Intercept is calculated for a female bank vole of average weight, captured from a 100+ year stand with average numbers of bank voles and other small mammals.

## Discussion

This study demonstrated that bank voles infected with Puumala hantavirus can be found commonly in young forests undergoing intensive management. However, they are less abundant than in mature forest stands 100+ years old. The probability of a bank vole being PUUV infected was positively related to concurrent bank vole abundance and negatively related to the abundance of other small mammals. Furthermore, bank voles inhabiting young forests were more likely to be PUUV infected than those in mature stands.

### Effects of Forestry on PUUV-infected Bank Vole Density

During most of the trapping periods within our four-year study, PUUV-infected bank voles were found in all forest age classes, being relatively common even in young stands. Hence, the managed boreal landscape of different aged forests poses no barrier to either bank voles or the spread of PUUV, unlike agricultural land surrounding forest patches in temperate Europe [19]–[22], [45]. As the stands in this study represented a wide range of age classes, it is unlikely that PUUV-infected bank voles would be scarce or absent from succession stages not covered by this study, i.e., 30–100 year old forests. Thus, we assume that the vast majority of Finnish forests (i.e., 77% of land cover) provide suitable habitat for bank voles and enable high connectivity among populations of hosts and viral strains. Therefore, we do not believe that boreal bank vole populations are sensitive to local stochastic extinction and colonization events, which can impair the persistence of pathogens in temperate host populations [19], [21], [46].

Although PUUV-infected bank voles were present in all forest age classes, they were 46–64% less abundant in young than in mature forest stands. Given that a larger number of infected animals likely indicates a larger number of bank voles shedding PUUV, mature forests may support a higher viral load and pose a higher risk of transmission to humans.

Whereas forest disturbance by agriculture is associated with low human NE incidence, disturbance due to intensive forest management is not. The disease is approximately three times more common in eastern and central than in south-western parts of Finland [47] yet the forest age structure is quite uniform across the country [23]. The most important reason for the epidemiological pattern seems to be the degree of cyclicity in voles. There is a steep gradient in snow cover from south-western to northern and eastern Finland that affects the community structure of prey and their predators. Consequently, the amplitude of vole population cyclicity increases from the southwest to the north [29], [48], [49]. As such, population peaks in the north likely cause a rapid increase in the number of PUUV-spreading bank voles [50]. In addition, south-western Finland is characterized by a higher proportion of agricultural and urban environments less frequented by bank voles.

### Factors Contributing to the Likelihood of a Bank Vole Being Infected with PUUV

Bank voles in 100+ year forests were less likely to be PUUV infected than their conspecifics in younger stands. This observation could be due to one or both of the following reasons; firstly, the lower bank vole abundance in young forests suggests they are less favorable habitats [25]. The lack of resources in young forests could reduce the condition of resident bank voles and thereby increase their vulnerability to pathogens. This explanation is supported by our observations of PUUV infection rates peaking in 25–30 year forests, which appear to be the least favorable habitat since they harbored the lowest number of bank voles during most of the study period. Secondly, environmental conditions in younger forests could favor the survival of PUUV outside the host. The highest infection rates in both spring and fall occurred in 25–30 year stands, where dense canopies maintained high humidity, lower temperature at ground level and blocked ultraviolet radiation. These environmental conditions could potentially prolong the period over which viral particles remain infective outside a host [9].

We observed a positive relationship between the probability of being PUUV infected and concurrent host population density. This result implies that transmission increases with bank vole density, either between infected and susceptible individuals or between susceptible individuals and an infectious environment. Several studies focusing on the PUUV hantavirus and bank voles have addressed the association between infection prevalence and host density [12], [19], [22], [37], [38], [51], [52], but positive relationships have only been found occasionally, and in most cases without time lags [12], [22], [52].

We found that abundance of other (non-host) small mammals was negatively correlated with the likelihood of a bank voles being PUUV infected during the breeding season. This result is consistent with other studies where hantavirus prevalence was negatively correlated with diversity [53]–[55], presence [56], or proportion [19], [57] (but see [52]) of non-host small mammals. However, we observed that an increasing number of non-host small mammals reduced the likelihood of a bank vole being infected when host density was constant (Table 2, Figure 3C). Therefore, we consider the association a true dilution effect in that the effect of non-hosts on pathogen prevalence occurs irrespective of host density, in contrast to an apparent one where non-hosts regulate host density. To date, a true dilution effect has been reported in three hantavirus studies [19], [55], [56] and has been hypothesized to take place when non-host species reduce intraspecific contact rates of hosts by altering their behavior [31]. Among Fennoscandian boreal rodents, *Microtus* spp. and *Myodes rufocanus* are competitively superior to the bank vole [27], [58], [59]. Shrews of genus *Sorex* have been considered competitively inferior or neutral [60], [61], but experimental data show that both field voles and common shrews can reduce the home range of breeding female bank voles [62]–[64]. Therefore, one might presume that in spring when bank voles are breeding, both the presence of non-host rodents or shrews could alter the behavior of bank voles, reducing their contact rate with PUUV-infected individuals or an infectious environment. In the fall, most bank voles were sexually inactive and therefore more tolerant of conspecifics [65]. As such, intraspecific contact rate and the probability of acquiring an infection may then be less affected by other species, which could explain why no dilution effect was observed in the fall.

In this study, and in several others concerning hantaviruses and their hosts [37]–[39], [52], [66], males were more often infected than females. This observation was limited to heavier, reproductive individuals. Regardless of whether these age- and sex-related differences result from behavioral or immunological factors, duration of exposure, or biased weights of pregnant females, we emphasize the need to control for these confounding factors when analyzing patterns of pathogen prevalence or infection probability.

We observed that young intensively managed forests are associated with lower bank vole and higher non-host densities. Both these characteristics of the small mammal community reduced the probability of a bank vole being infected with PUUV. On the other hand, we also observed a higher probability of bank voles being infected when trapped in young rather than old forest stands, i.e., a contrary effect that was not attributable to either host or non-host density. In conclusion, we believe that the combined effect of these three factors caused a homogeneous prevalence of PUUV in bank voles among all forest age classes (Figure 1B).

### Conclusions

All forest age classes harbored PUUV-infected bank voles in the cyclic peak phase, indicating that all forest habitats, be they managed or old-growth, are potential sources of NE infection for humans. In contrast to the agricultural landscape in temperate Europe, intensive management of Fennoscandian forests does not create significant barriers to the spread of bank voles and PUUV. We found bank voles trapped in young forests were more likely infected, but the mechanisms of this association require further investigation. We also found infection probability to be host density dependent. Furthermore, we observed a statistically significant dilution effect in breeding animals when host density was controlled for, but not among non-breeding individuals in the fall. Based on our findings, it seems likely that the species composition of the small mammal community could indeed play a role in PUUV infection dynamics.

## Supporting Information

Figure S1 **Representative photographs of the four studied forest age classes.**
(TIF)
