# Supplementary figures and images for: Environmental Change and Disease Dynamics: Effects of Intensive Forest Management on Puumala Hantavirus Infection in Boreal Bank Vole Populations
Source: PLoS One. 2012 Jun 20;7(6):e39452. doi: 10.1371/journal.pone.0039452 (PMC3380007; doi:10.1371/journal.pone.0039452)

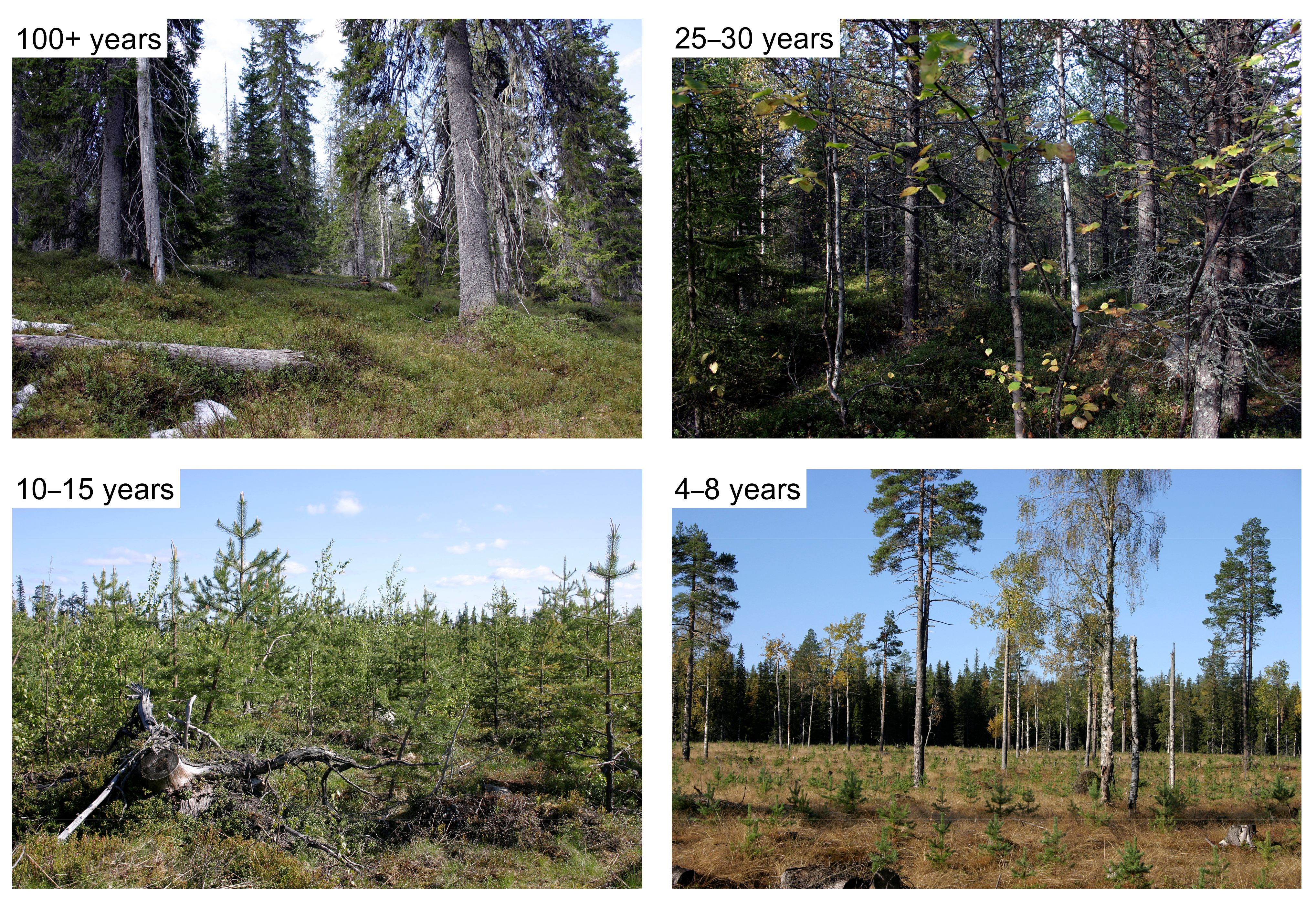

Supplement: Figure S1 — Representative photographs of the four studied forest age classes. (TIF) [file pone.0039452.s001.tif]
